# Variability in MRI vs. ultrasound measures of prostate volume and its impact on treatment recommendations for favorable-risk prostate cancer patients: a case series

**DOI:** 10.1186/1748-717X-9-200

**Published:** 2014-09-09

**Authors:** Yonina R Murciano-Goroff, Luciant D Wolfsberger, Arti Parekh, Fiona M Fennessy, Kemal Tuncali, Peter F Orio, Thomas R Niedermayr, W Warren Suh, Phillip M Devlin, Clare Mary C Tempany, Emily H Neubauer Sugar, Desmond A O’Farrell, Graeme Steele, Michael O’Leary, Ivan Buzurovic, Antonio L Damato, Robert A Cormack, Andriy Y Fedorov, Paul L Nguyen

**Affiliations:** Brigham and Women’s Hospital, Radiation Oncology, 75 Francis Street, Boston, MA 02115 USA; Dana-Farber Cancer Institute, 50 Brookline Avenue, Boston, MA 02115 USA; Harvard Medical School, 25 Shattuck Street, Boston, MA 02115 USA; Cancer Center of Santa Barbara, 519 W. Junipero Street, Santa Barbara, CA 93105 USA

**Keywords:** Prostate volume, Favorable-risk prostate cancer, Brachytherapy, Active surveillance, MRI, Ultrasound

## Abstract

**Background:**

Prostate volume can affect whether patients qualify for brachytherapy (desired size ≥20 mL and ≤60 mL) and/or active surveillance (desired PSA density ≤0.15 for very low risk disease). This study examines variability in prostate volume measurements depending on imaging modality used (ultrasound versus MRI) and volume calculation technique (contouring versus ellipsoid) and quantifies the impact of this variability on treatment recommendations for men with favorable-risk prostate cancer.

**Methods:**

We examined 70 patients who presented consecutively for consideration of brachytherapy for favorable-risk prostate cancer who had volume estimates by three methods: contoured axial ultrasound slices, ultrasound ellipsoid (height × width × length × 0.523) calculation, and endorectal coil MRI (erMRI) ellipsoid calculation.

**Results:**

Average gland size by the contoured ultrasound, ellipsoid ultrasound, and erMRI methods were 33.99, 37.16, and 39.62 mLs, respectively. All pairwise comparisons between methods were statistically significant (all p < 0.015). Of the 66 patients who volumetrically qualified for brachytherapy on ellipsoid ultrasound measures, 22 (33.33%) did not qualify on ellipsoid erMRI or contoured ultrasound measures. 38 patients (54.28%) had PSA density ≤0.15 ng/dl as calculated using ellipsoid ultrasound volumes, compared to 34 (48.57%) and 38 patients (54.28%) using contoured ultrasound and ellipsoid erMRI volumes, respectively.

**Conclusions:**

The ultrasound ellipsoid and erMRI ellipsoid methods appeared to overestimate ultrasound contoured volume by an average of 9.34% and 16.57% respectively. 33.33% of those who qualified for brachytherapy based on ellipsoid ultrasound volume would be disqualified based on ultrasound contoured and/or erMRI ellipsoid volume. As treatment recommendations increasingly rely on estimates of prostate size, clinicians must consider method of volume estimation.

## Background

Eligibility for both brachytherapy [1] and active surveillance in men with favorable-risk prostate cancer is partially dependent on determination of prostate volume [2]. Desired volumes for brachytherapy are typically cited as ≥20 mL [3–5] and ≤60 mL [1], while classification into the “very low-risk” group that is often offered active surveillance requires a PSA density of ≤0.15 ng/dl, calculated on the basis of measured prostate volume [2]. Despite the dependence of treatment recommendations on prostate volume, the method by which the volume should be estimated is rarely specified in guidelines.

Three major techniques are currently in widespread use to determine prostate size. From a transrectal ultrasound (TRUS), volume can be estimated via the traditional ellipsoid estimation based on the height (H), width (W), and length (L) of the prostate, using the formula: H × W × L × 0.523. Alternatively, the same TRUS can be contoured on multiple axial slices (of thickness typically between 2.5 mm and 5 mm) during a brachytherapy volume study and then integrated in 3D space to generate a contoured volume estimate. Finally, MRIs are being increasingly used to stage prostate cancer and commonly report volume estimates based on the ellipsoid formula.

There have been some studies examining the differences between the volume estimates of these various modalities [6–11], but currently it is unknown whether differences in the methods of volume determination could translate into differences in treatment recommendations for favorable-risk patients.

Therefore, our study aimed to assess whether differences in prostate volume as estimated by use of the ellipsoid formula based on TRUS measurements, contouring on TRUS, and use of the ellipsoid formula based on measurements from endorectal coil MRI (erMRI) would have implications for the determination of the eligibility of men with favorable-risk prostate cancer for either brachytherapy or active surveillance.

## Methods

Institutional Review Board approval was obtained from the Dana-Farber Cancer Institute. The study examined 70 men who presented consecutively to one of the authors (PLN, Dana-Farber Cancer Institute) for consideration of brachytherapy for the treatment of favorable risk prostate cancer and had three estimates of prostate volume from the ellipsoid TRUS method, contoured TRUS method, and ellipsoid erMRI method with available MRI dimensions.

From an initial pool of 90 consecutive men, eight men who received Dutasteride or Androgen Deprivation Therapy were excluded from consideration owing to the role of these therapies in altering prostate size, as was the one individual who presented for consideration of salvage, rather than first-line, therapy. Patients who did not have all three measures of volume or for whom erMRI height, width, and length measures were not available (n = 11) were excluded, leaving 70 patients for whom all data was available.

All individuals included in the analysis underwent prostate volume studies between August 2009 and June 2011. Volume studies typically included 3 separate volume estimates made at the time of presentation, and typically occurred on the same day. Ultrasound studies were performed in the dorsal lithotomy position with a BK probe ® (BK Medical, Peabody, MA) connected to a Nucletron FIRST system with SPOT-PRO v3.1 ™ (Nucletron, an Elekta company, Elekta AB, Stockholm, Sweden) for acquiring ultrasound information in the sagittal plane and generating a 3D reconstruction for axial contouring. Volume was evaluated according to the ellipsoid method, in which height, width, and length were used to calculate overall gland volume based on the standard BK ultrasound prostate volume formula, namely H × W × L × 0.523. Contouring was done on the reconstructed ultrasound axial slices (2.5 mm thickness), and was also used to estimate volume based on an integration of the slices by the FIRST system (Nucletron, an Elekta Company). Additionally, patients underwent a 3Tesla multiparametric T2 erMRI, from which prostate volume was calculated using the standard MRI ellipsoid formula of H × W × L × 0.523.

Desired size for brachytherapy was taken to be between ≥20 mL [3–5] and ≤60 mL [1]. PSA densities were calculated using the latest PSA values available at the time of consideration for brachytherapy. Absolute PSA was divided by measured gland volume to obtain density. In keeping with the National Comprehensive Cancer Network (NCCN) Guidelines ® (NCCN, Fort Washington, PA), PSA density of ≤0.15 was regarded as a cut-off for the “very low risk” group that is typically offered active surveillance [2].

Prostate volumes and PSA densities obtained using the different calculations of gland size were compared using standard, paired t-tests carried out with STATA software. P-values of less than 0.05 were said to reflect statistical significance.

## Results

### Variation in Prostate Size by Method of Estimation

**Figure 1:**
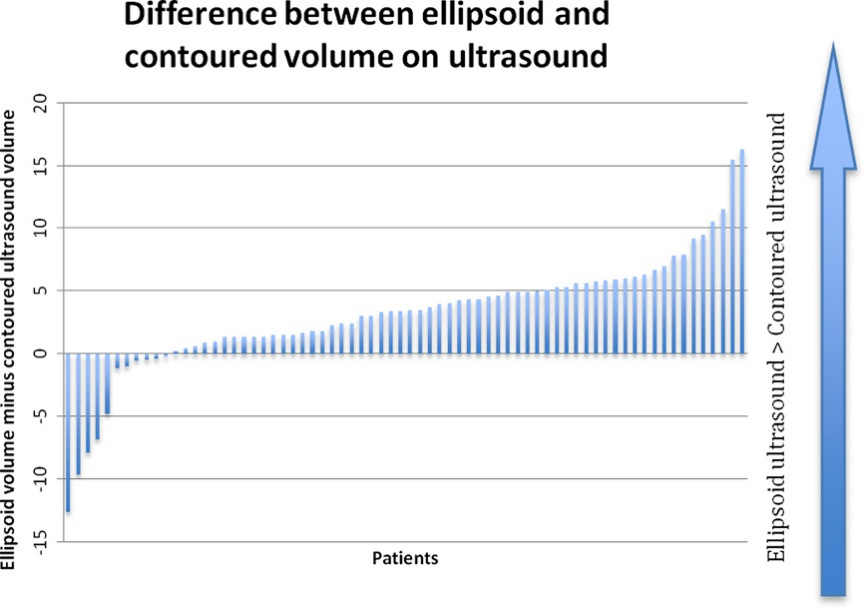
**Differences in prostate volume measures using ellipsoid ultrasound versus contoured ultrasound.**

**Figure 2:**
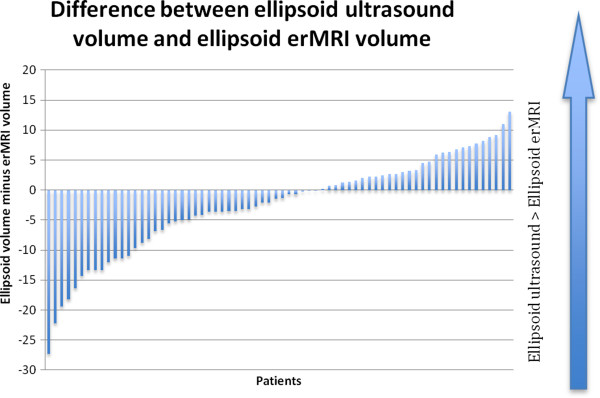
**Differences in prostate volume measures using ellipsoid ultrasound and erMRI.**

Estimates of prostate volume differed when disparate scanning modalities were used. Average gland size calculated by the contoured ultrasound, ellipsoid ultrasound, and erMRI methods were 33.99, 37.16, and 39.62 mLs, respectively. The difference between the average volume obtained on contoured ultrasound and that obtained on ellipsoid ultrasound was significant (p = 0.00) (Figure 1) as was the difference between average gland size on contoured ultrasound and erMRI (p = 0.00). The comparison between average ellipsoid and erMRI prostate volumes was also significant (p = 0.01) (Figure 2).

### Impact of Estimation Method on Qualification for Brachytherapy

Different numbers of patients fit the prostate volume eligibility criteria for brachytherapy when disparate scanning modalities were used to calculate gland size. Of the 70 patients examined, all except 4 were eligible for brachytherapy when prostate volumes were calculated using the ellipsoid ultrasound method (desirable volume ≥20 mL and ≤60 mL). Of the 4 patients deemed ineligible on ellipsoid ultrasound measures, 2 (50%) met eligibility criteria for brachytherapy based on erMRI measures of volume.

Among the remaining 66 patients deemed anatomical candidates for brachytherapy based on ellipsoid ultrasound measures (size ≥ 20 mL and ≤60 mL), 22 (33.33%) did not qualify for brachytherapy based on volumes measured using one or both of the other scanning modalities. In particular, 16 patients would not have qualified on erMRI (6 patients <20 mL, 10 patients >60 mL) and 10 patients would not have qualified based on contoured ultrasound measures (7 patients <20 mL, 3 patients >60 mL).

**Figure 3:**
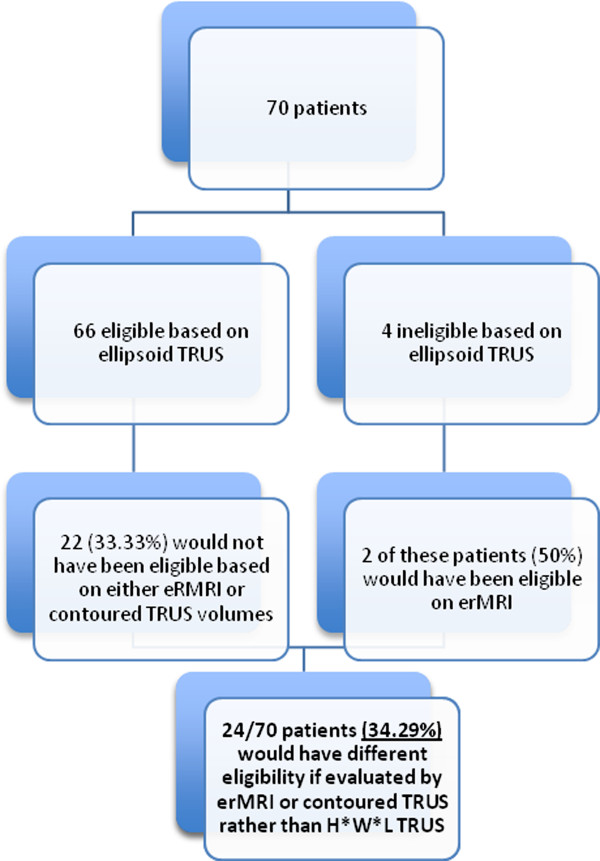
**Eligibility for brachytherapy.**

6 of the 66 patients who were considered candidates for brachytherapy as determined by the ellipsoid ultrasound technique for measuring prostate volume had prostates <20 mL on erMRI (9.09%) and 10 patients had glands measuring >60 mL on erMRI (15.15%). 7 of the 66 patients who were anatomically good candidates for brachytherapy on ellipsoid ultrasound had prostate sizes <20 mL (10.61%) on contoured ultrasound, and 3 patients had volumes >60 mL (4.55%).In total, the management of 24/70 patients (34.29%) would have changed if patient prostate size had been measured by a method other than ellipsoid TRUS (Figure 3).

### Impact of Estimation Method on PSA Density

Each of the different scanning modalities was also used to calculate a PSA density. Using ellipsoid ultrasound volumes, 38 patients (54.28%) had a PSA density ≤ 0.15 ng/dl, which is the cut-off for the very low-risk group that is commonly offered active surveillance [2]. When volume was determined by erMRI, 34 patients (48.57%) were deemed to have PSA density ≤ 0.15 ng/dl, while 38 patients (54.28%) were categorized as having PSA density ≤ 0.15 ng/dl using contoured ultrasound estimates of volume.

## Discussion

Determination of eligibility for brachytherapy generally relies on prostate volume based criteria [1, 3–5], but there are no agreed upon guidelines for how volume should be measured. Studies have examined the potential impact of large gland size on accurate dosimetry [12], pubic arch interference [13, 14], as well as urinary retention [12, 14–16]. The technical feasibility of accurate dosimetry and effective outcomes [6, 13, 17–19] in men with smaller prostates who receive brachytherapy has also been analyzed. Previous studies of the impact of prostate volume on brachytherapy dosimetry and complication rates have used numerous methods to determine volume, including contoured TRUS [14–16, 18, 20], ellipsoid TRUS [21], and MRI [22, 23].

In our study, the ellipsoid ultrasound and ellipsoid erMRI methods overestimated TRUS contoured volume by an average of 9.34% and 16.57%, respectively. These results reflect the fact that no prostate is an exact ellipsoid, and while formulas based solely on height, width, and length are very convenient, they can only approximate the volume. Contouring on thinly cut axial slices can account for variability in prostate shape from the base to the apex [24].

While previous studies of volume estimation in brachytherapy candidates have shown smaller differences between TRUS and MRI-based estimations than our findings [7–9], most studies have compared TRUS to contoured MRI, which is less clinically practical than using an ellipsoid estimation of volume on MRI. The one study to compare volumes derived from contouring on TRUS with ellipsoid MRI volumes in 15 patients who were not receiving hormone therapy found a correlation between volumes of r = 0.83 [10].

In our study, volumes estimated by erMRI were larger than those calculated on TRUS by either contouring or the ellipsoid method. These results may reflect the fact that MRI enables better soft tissue density determination, leading to greater maximal dimension estimates and inflating calculated volume estimates. It may also reflect the fact that on MRI, transagittal cuts are sometimes used for height estimates, rather than the transaxial cuts used on TRUS. Previous work has demonstrated that such a distinction in means of measuring height can alter volume estimates [25].

Our study is unique in that it may be the first to quantify the impact of differences in volumes derived from disparate scanning modalities on decision-making surrounding candidacy for brachytherapy. Of patients who qualified for brachytherapy based on the commonly used ellipsoid ultrasound technique for volume estimation, 33.33% would not have qualified for brachytherapy were they assessed using ultrasound contoured and/or erMRI estimated volumes. Management of 34.29% of patients would have changed had volume been determined by a method other than ellipsoid TRUS.

We also examined the impact of different volume measures on patients’ eligibility for active surveillance based on standard PSA density criteria (desired PSA density ≤ 0.15 ng/dl), and found that while 54.28% of patients were eligible using ellipsoid TRUS measures, 54.28% and 48.57% were eligible using erMRI and contoured ultrasound measures, respectively. The one previous study of active surveillance eligibility demonstrated that of five patients who were initially enrolled in an active surveillance program but were found to be ineligible based on contoured MRI, one patient (20%) would still have been eligible based on ellipsoid TRUS [11]. Our study differs in our use of ellipsoid rather than contoured MRI as well as the diversity of our cohort, which was not limited solely to patients already enrolled in an active surveillance program.

The practical implications of our study are that clinicians should be aware that volume estimates of prostate size can vary significantly depending on how the estimate was obtained, and so when treatment decisions are highly dependent on prostate size, it may be worthwhile to consider the source of the estimate and how close the estimate sits to the boundary of a clinical guideline. For example, a patient with a prostate size of 70 cc estimated on a prostate MRI who is otherwise a great candidate for brachytherapy might only have a volume of 55 cc on a contoured ultrasound.

While our study is the largest to examine differences in prostate volume among brachytherapy candidates using disparate imaging modalities, it also has certain limitations. First, all cases were drawn from a single medical center with imaging carried out and interpreted at the center. Secondly, the ultrasound and erMRI scans done on our patients were not all carried out at precisely the same time, leading to possible confounding by bladder filling. However, scans were typically performed on the same day and endorectal coil was used in an effort to improve the accuracy of all MRI scans [26, 27].

## Conclusions

Our results point to significant differences in treatment recommendations depending on which imaging modality is used. In patients in whom prostate volume measurements or PSA density numbers are borderline for eligibility for brachytherapy or active surveillance, clinicians may wish to consider the possibility that disparate scanning modalities could yield different volume estimates. As treatment recommendations increasingly depend on prostate size, clinicians will need to take into account the method by which volume was estimated in determining treatment plans.

## Abbreviations

erMRI: Endorectal coil MRI
TRUS: Transrectal ultrasound
H: Height
W: Width
L: Length.
